# Psychiatry

**Published:** 1905-07-08

**Authors:** 


### Progress in Medicine and Surgery.

### PSYCHIATRY.

(*Continued from page* 245.)

**Types of Alcoholic Insanity and Dipsomania.**—An interesting series of contributions was made and discussed at a recent meeting of the Boston Society of Psychiatry and Neurology on this subject. Dr. H. W. Michell in the opening contribution deals with the investigation of 148 male alcoholic cases of insanity, excluding two dipsomaniacs who for long intervals (months or even a year or two) showed no psychical symptoms, and only had their periodic but short outbreaks lasting one to three or four or six days at a time. These 148 cases were all admitted to the Danvers Insane Hospital during five years (1889–1904). They were classified as follows:—(*a*) Delirium tremens; (*b*) hallucinatory alcoholism, acute and subacute; (*c*) alcoholic delusional insanity (chronic); and (*d*) alcoholic dementia (the last stages of mental deterioration and decay). There were, he said, two cases of Karsakoff's disease of alcoholic origin among these. He found that among cases of delirium tremens, "nearly all recovered without passing into the graver forms of alcoholic insanity." His prognosis in cases of acute alcoholic hallucinations was "good for the attack, but relapses due to renewal of drinking habits were common. These cases were characterised by active auditory and visual hallucinations, the former predominate, with little disturbance of consciousness, and transitory development of delusions based upon the hallucinations." In the case of subacute hallucinatory insanity there were auditory, visual, olfactory, and tactile hallucinations, with more prolonged genesis of delusions, largely dependent on hallucinations. "Periodic relapses were frequently seen in these cases while in hospital, with practically normal reaction in the intervals. Relapses were common after leaving hospital, and there was frequent and permanent mental deterioration. The term alcoholic delusional insanity was not used for cases showing paranoia-like genesis of delusions. Delusions were sometimes elaborated from hallucinations, but in many cases were not associated with any hallucinatory disturbance. Ideas of marital infidelity, poisoning and persecution were most common. "A tendency to chronicity was observed in these cases, only a small proportion recovering. Alcoholic dementia included cases showing dementia as the principal symptom, and this occurred as the result of many years of more or less continued use of distilled liquors. Permanent mental deterioration was seen in all the cases studied." Among the alcoholic psychoses seen, many clinically resembled general paralysis, the diagnosis being possible only after prolonged observation for weeks. Ten per cent. of the alcoholic cases had one or more epileptiform convulsions. Suicidal attempts and acts of violence were common and were seen in relation to hallucinations. An insane or alcoholic ancestry was common, and made prognosis bad. Somatic and grandiose delusions with alteration of personality were unfavourable symptoms in delusional cases. Periodic drinking was more common in delirium tremens and hallucinatory insanity, daily drinking was associated with and led to delusional insanity and dementia. In the discussion which followed the above paper, Dr. Woodbury said that probably 90 per cent. of first attacks of delirium tremens recovered. Dr. Stedman alluded to the rare category of cases known as true dipsomania. These, he said, were periodic and irresistible impulses of drink craving; the intervals were periods of perfect sobriety. The victim struggled against the oncoming craving, but succumbed through inadequate power of resistance to it. "When seized by the impulse, a business man, otherwise prudent and scrupulous, would suddenly leave his office, or a father his family while at church, for the nearest resort, where he drank till his craving was appeased and then returned home overwhelmed with remorse." This impulsive, morbid, irresistible craving, independent of, and in conflict with the will was a symptom of hereditary neuropathy. It was distinguished from drunkenness (which was mainly a vicious and depraved habit), and was really allied to those forms of obsessional and impulsive insanity known as "manias" and "phobias," especially as regards periodicity. They were essentially rare cases. Dr. Albert M. Barrett referred to the views enunciated by Gaup that dipsomania was a "form of epilepsy." Under dipsomania he placed those cases of periodic drinking in which, after a prodromal emotional agitation—a mental struggle within the ego—there supervened an irresistible impulse to alcoholic excess. This lasted for days, when the normal state gradually returned. Gaup described three sub-groups: (*a*) Pure dipsomania, in which the period of drinking came on after an emotional depression; (*b*) cases of periodic depression without inclination for drink, but which were characterised by the presence of "typical epileptic symptoms," such as convulsions, or various forms of psychical epilepsy; (*c*) cases in which dipsomaniacal attacks were combined with post-epileptic "dreamy states" of consciousness, "epileptic vertigo," or fits. Gaup concludes that the attacks of depression in dipsomania are parallel in nature to, if not identical with, those of epilepsy, and that dipsomania is a neurosis closely allied to epilepsy; "in fact one of the many forms of epilepsy." As many cases of dipsomania are often associated with criminal acts, the forensic importance of such conclusions as those of Gaup will be apparent. Dr. Knapp said that in the wards and clinics of the City Hospital at Boston the cases of delirium tremens, hallucinatory alcoholism, and alcoholic dementia were fairly common, while paranoid forms were quite rare; transitional cases occurred between alcoholic hallucination and delirium tremens. Dr. Mitchell, in reply, said that cases of alcoholic pseudo-paresis were fairly common. They had sluggish pupils, but pupils that reacted to light. Inequality of pupils and failure of the light reflex was practically limited to syphilitic cases, but prolonged observation was required. The delusional cases in his series amounted to nearly 20 per cent. Parental intemperance was common, but exact figures could not be given because of the foreign element. The milder form of alcoholic psychosis occurred in early life, in association with periodic drinking habits, and this included also delirium tremens. Alcoholic dementia occurred in persons past middle life and always showed a history of hard and heavy drinking.

[8] Jour. of Nerv. and Ment. Dis., December 1904.

